# Identification and bioinformatic analysis of *Aux/IAA* family based on transcriptome data of *Bletilla striata*

**DOI:** 10.1080/21655979.2019.1692610

**Published:** 2019-11-25

**Authors:** Houbo Liu, Lin Li, Chun Li, Ceyin Huang, Yanni ShangGuan, Ronghui Chen, Shiji Xiao, Weie Wen, Delin Xu

**Affiliations:** a Department of Cell Biology, Zunyi Medical University, Zunyi, China; b Sesame Research Institute, Chinese Academy of Agriculture Sciences, Zheng Zhou, China; c Department of Pharmacy, Zunyi Medical University, Zunyi, China

**Keywords:** *Bletilla striata*, genome-wide, *Aux/IAA* gene family, bioinformatic, evolutionary analysis, EST-SSR

## Abstract

Auxin/Indole-3-Acetic Acid (*Aux/IAA*) genes are involved in auxin signaling pathway and play an important role in plant growth and development. However, many studies focus on *Aux/IAA* gene families and much less known in *Bletilla striata*. In this study, a total of 27 *Aux/IAA* genes (BsIAA1-27) were cloned from the transcriptome of *Bletilla striata*. Based on a phylogenetic analysis of the Aux/IAA protein sequences from *B. striata, Arabidopsis thaliana* and *Dendrobium officinale*, the *Aux/IAA* genes of *B. striata* (*BsIAAs*) were categorized into 2 subfamilies and 9 groups. While *BsIAAs* were more closer to those of *D. officinale* compared to *A. thaliana*. EST-SSR marker mining test showed that 4 markers could be stably amplified with obvious polymorphisms among 4 landraces. Our results suggested that *BsIAAs* were involved in the process of tuber development and provided insights into functional roles of *Aux/IAA* genes in *B. striata* and other plants.

## Graphical Abstract

**Figure:**
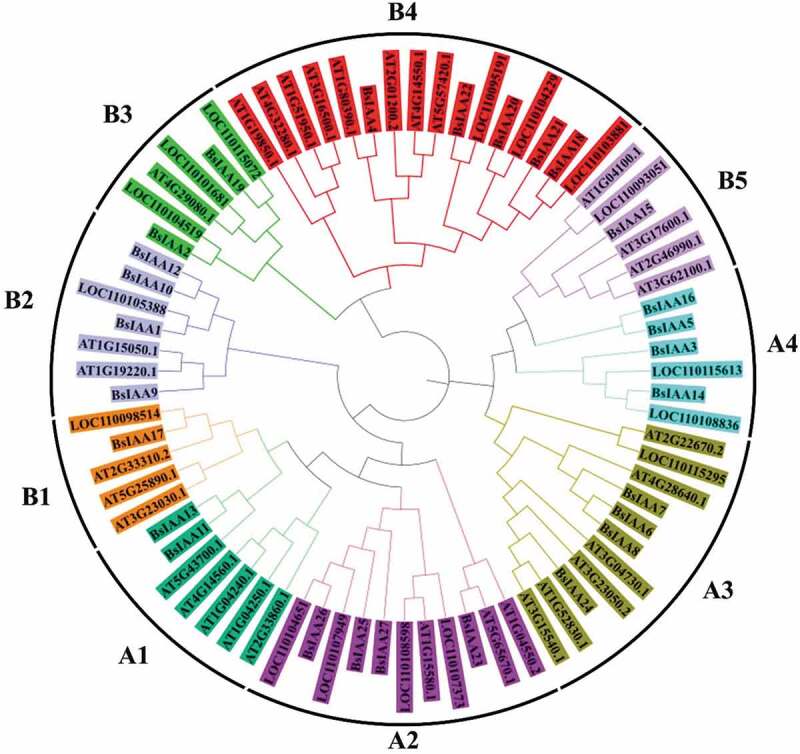

In this paper, we identified the 27 members of the *Aux/IAA* gene family which can be divided into 2 groups and 9 subfamilies. The similar branches of Aux/IAA proteins had the same or similar motifs, such as genes between *BsIAA10* and *BsIAA12, BsIAA11* and *BsIAA13, BsIAA18* and *BsIAA20*, indicating that *Aux/IAA* proteins were conserved. Among them, *BsIAAs* were more closer to those of *D. officinale* compared to *A. thaliana*.

## Introduction

Auxin is the first discovered plant hormone playing an important role in plant growth and development [1, 2]. It consists of a group of molecules with an anthracene ring and is a commonly used signal chemical in plants. Auxin regulates cell division and elongation of plants, and organ development of cells and whole plants [2]. Growing evidence indicates that auxin, either alone or together with other hormones, is involved in plant responses to environmental stimuli, including drought, cold, and salt [3,4]. *Aux/IAA, SAUR* and *GH3* are the three major gene families of early auxin response, which are responsive to early auxin induction [5]. As an important regulatory gene in the auxin signaling pathway, *Aux/IAA* gene has become a hot topic in recent years. Its protein sequence has four conserved domains. Domain I has transcriptional inhibition, II acts on the stability of its protein, III and IV are responsible for protein dimerization [6]. This protein and the transcription factor ARF (auxin response factor) family can form heterodimers. Under the action of auxin, the protein of Aux/IAA is degraded, which triggers the expression of downstream genes of auxin signaling pathway [7,8]. Since the first *Aux/IAA* gene was cloned, 32 *Aux/IAA* genes have been identified from *Arabidopsis* genome-wide analysis, and a large number of *Aux/IAA* family members have also been identified in other plants, including eucalyptus [9], cucumber [10], maize [11], soybean [12] and so on. Genome-wide analysis showed that the members of the *Aux/IAA* gene family had different biological functions. In *Hedychium coronarium, HcIAA2* and *HcIAA4* play important roles in its floral scent formation [13]. In *Medicago truncatula, MtIAA6* and *MtIAA7* exhibit root-specific expressions and *MtIAA9* shows higher expression level in flower [14]. In *Dendrocalamus sinicus, DsIAA3, DsIAA4, DsIAA15* and *DsIAA20* may be important for regulating shoot development [15]. In conclusion, these studies indicate that the *Aux/IAA* gene family is involved in the regulation of plant growth and development and response to multiple signal transduction pathways. The analysis of *Aux/IAA* gene family is not only helpful to elucidate the molecular mechanism of auxin metabolism and signal transduction, but also can be used in plant genetic research.

*Bletilla striata* is a perennial herbaceous temperate plant of Orchidaceae with many significant values on medicine, ornamental and so on [16]. And the secondary metabolites are the important medicinal components of it, so it is necessary to analyze the genes related to the synthesis of the secondary metabolites. However, there are few reports on its growth regulation and secondary metabolite synthesis. And there is no report on the systematic identification and analysis of *Aux/IAA* gene family in *B. striata* now. In consequence, this study intended to analyze the *Aux/IAA* gene family members of *B. striata* by bioinformatics methods based on the entire transcriptome data of developmental organs covering the entire growth phase in the early stage, and design specific molecular markers based on their sequences. It may provide a theoretical basis for the related utilization of *Aux/IAA* genes in *B. striata*, and provide clues for the study of functional characteristics of auxin-responsive genes.

## 1 Materials and methods

### 1.1 Materials

The *B. striata* seeds were purchased from a local farmer planting medicinal herbs in Zheng’an County (28°56′N, 107°43′E), Guizhou Province, China, on October 20^th^, 2015. The seeds were germinated and formed into seedlings by tissue culture, and the refined seedlings were transplanted to the test site. The protocorms, whole seedlings before seedling transplanting, whole seedlings after transplanting for 2 months, whole plants after transplanting for 1 year (including roots, stems, leaves, flowers, and seeds for successful pollination) were randomly collected for total RNA extraction. Then after the detection, the qualified RNA samples were pooled with the same amount for the subsequent transcriptome sequencing [17].

### 1.2 Methods

#### 1.2.1 Transcriptome assembly

Using the Illumina HiSeq sequencing 2000 platform to conduct high-throughput sequencing of *B. striata*.And the resulting data were assembled by de novo using Trinity software to finally obtain the transcriptome data set of *B. striata*’s single gene sequence.

#### 1.2.2 Aux/IAA *gene family identification*

The sequences of *Aux/IAA* were obtained for identify the conserved domains. *Aux/IAA* genes of *Arabidopsis (AtIAAs)* were screened out by querying against the TAIR (The *Arabidopsis* Information Resource, http://www.arabidopsis.org/). Local tblastn search of *B. striata* proteomes by using Bioedit software (score value≥100 and evalue≤e^−10^) [18]. Selecting the assembled data of our group as the search database, and using the 29 protein sequences of *Arabidopsis* as query [19]. Using the online software Pfam and NCBI blast to screen for candidate sequences. All obtained protein sequences were examined for the presence of *Aux/IAA* (PF02309) domains by using the Hidden Markov Model of Pfam, SMART (http://smart.embl-heidelberg.de/) and InterPro (http://www.ebi.ac.uk/interpro/) tools.

#### 1.2.3 *Bioinformatics analysis of proteins of* Aux/IAA *genes*

To predict the coding sequences, we first applied ORF finder (https://www.ncbi.nlm.nih.gov/orffinder/) to predict the Open Reading Frame (ORF) of unigenes successfully matched by BLAST. Then, the basic physicochemical properties such as molecular weight, isoelectric point, and instability coefficient of each nucleotide sequence of *BsIAAs* were predicted by the online software ProtParam (http://web.expasy.org/protparam/). The subcellular localization of *BsIAA* proteins was analyzed using the online software Plant-mPLoc server (http://www.csbio.sjtu.edu.cn/bioinf/plant-multi/#). The secondary structure was analyzed using the online server SOPMA (https://npsa-prabi.ibcp.fr/cgi-bin/npsa_automat.pl?page=npsa_sopma.html).

**Figure 1.**
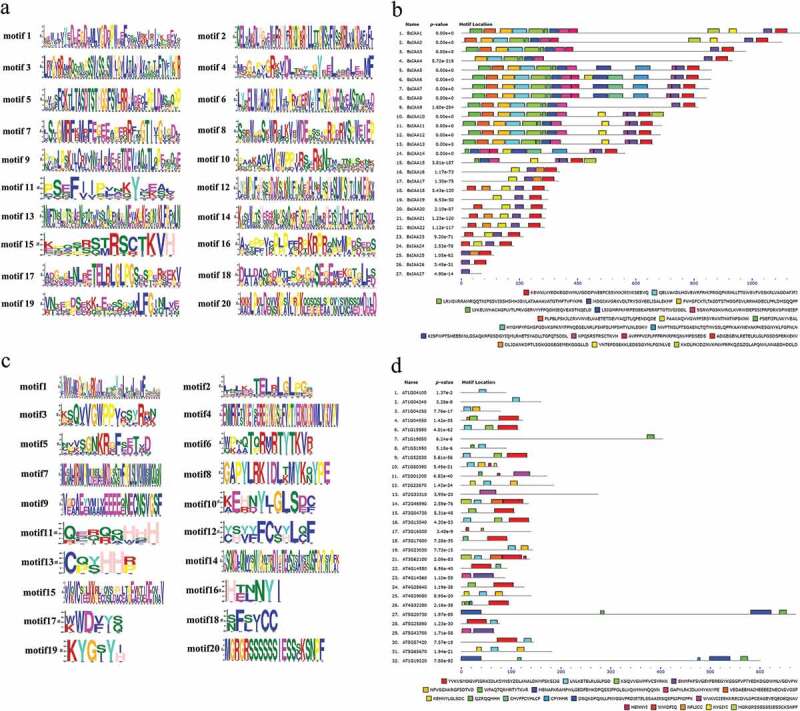
Conservative motif of *B. striata*(a,b) and *A. thaliana*(c,d).

**Figure 2.**
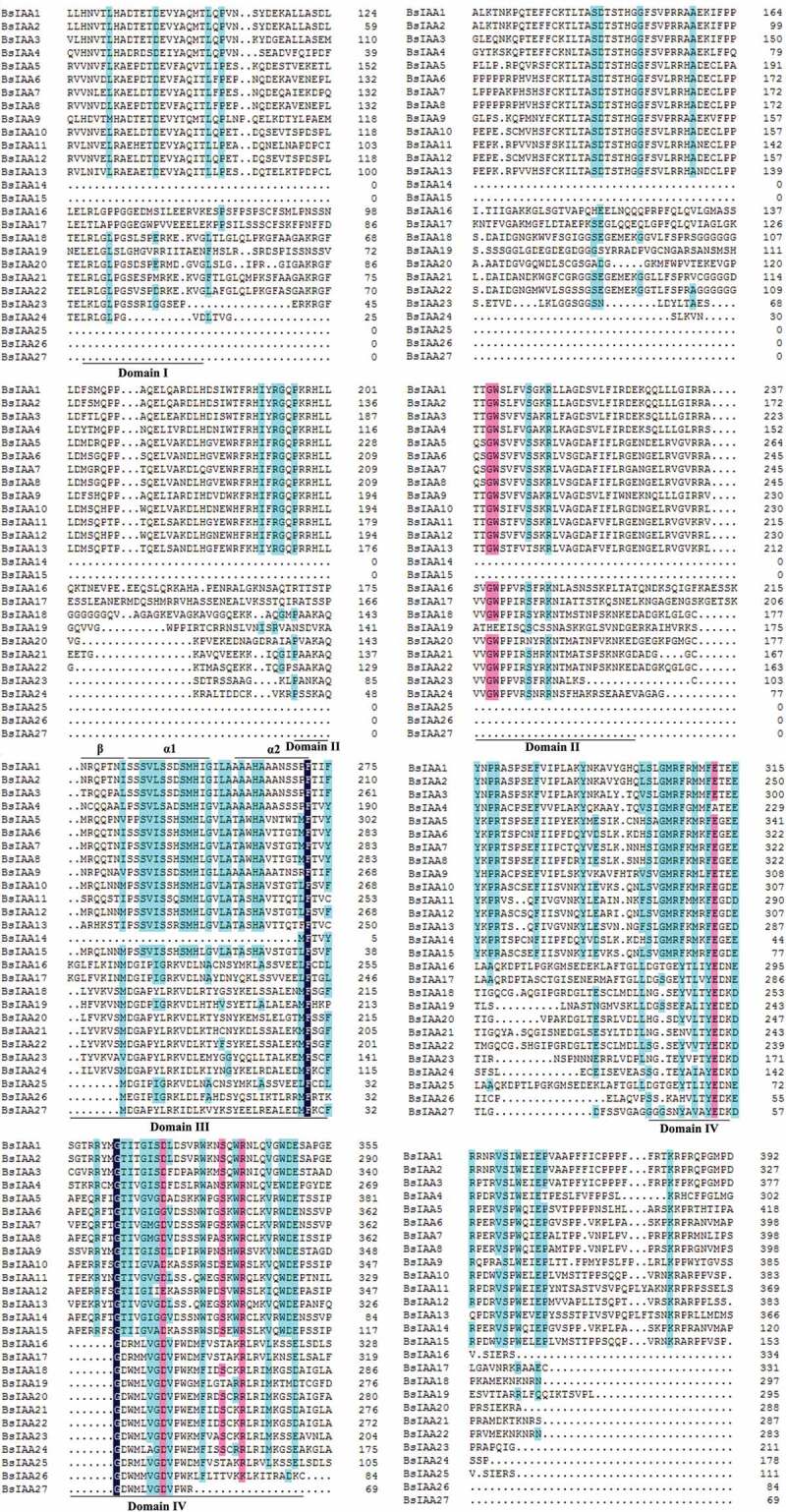
Multiple sequence alignment of *BsIAAs.*

Motif organization of *BsIAAs* and *AtIAAs* proteins was investigated by MEME5.0.5 (Figure 1). The maximum motif width was 50, the number of motifs was 20 [20] and the other parameters were default values. The *BsIAAs* protein conserved domains were aligned using DNAMAN software (Version 9) (Figure 2).

#### 1.2.4 *Construction of phylogenetic tree of* Aux/IAA *family of* D. candidum *and* A. thaliana

Multiple sequence alignments were generated using ClustalW in MEGA 7.0 with defaulted parameters. Phylogenetic tree was generated using neighbor-joining method with 1000 bootstrap test by MEGA 7.0 software.

#### 1.2.5 EST-SSR detection and verification

The plant samples of *B. striata* were collected from provinces of Sichuan, Chongqing, Guizhou and Anhui, China, for extracting genomic DNA according to the method of CTAB [21]. The extracted DNA was diluted to 50 ng/μL and stored at −40°C for EST-SSR detection.

The EST-SSR markers were detected, developed and verified through the following approaches. The online software NWISRL (https://ssr.nwisrl.ars.usda.gov/) was applied to detect EST-SSR sites of each *BsIAA* sequence with default parameter values. Then, primers of each site were designed using DNAMAN program. Subsequently, PCR amplification was used to verify the results of PAGE detection. The PCR reaction volume was 10 ul, containing 15 ng of template DNA, 6 uL 2× PCR MIX, 0.75 uL of each primer, and 1 uL of ddH_2_O. The amplification conditions were: pre-denaturation at 95 °C for 5 min; denaturation at 95 °C for 30 s, annealing at 58 °C for 30 s, extension at 72 °C for 60 s, 34 cycles, and extension at 72 °C for 5 min. The amplified product was separated by a 10% polyacrylamide gel. The electrophoresis apparatus was a PowerPac type with steady-state electrophoresis apparatus. The constant voltage was set to 150 V and electrophoresis was performed for 150 min. After silver nitrate staining, the bands were observed and photographed.

## 2 Results

### 2.1 Full transcriptome data construction

After assembling the RNA-seq data of all existing Pair ends by using Trinity software, the following data were obtained (Table 1). The sequencing approach obtained 106,054,784 clean reads (SRA database accession number: SRR7058048) by using an Illumina platform, and the reads were assembled into 134,900 unigenes by the Trinity package [17].

**Table 1.** *B. striata* full transcriptome data construction.

BioProject	Run	ReleaseDate	Bases	AvgLength	size_MB	Experiment
PRJNA451494	SRR7058048	2018/4/23	1.33E+10	250	6324	SRX3989034
PRJDB5866	DRR099052	2018/9/6	3.03E+09	202	1847	DRX092539
PRJDB5866	DRR099053	2018/9/6	2.38E+09	202	1448	DRX092540
PRJDB5866	DRR099054	2018/9/6	2.52E+09	202	1536	DRX092541
PRJDB5866	DRR099055	2018/9/6	2.53E+09	202	1546	DRX092542
PRJDB5866	DRR099056	2018/9/6	2.25E+09	202	1373	DRX092543
PRJDB5866	DRR099057	2018/9/6	2.59E+09	202	1584	DRX092544

### 2.2 *Identification of* Aux/IAA *gene families and analysis of protein characteristics*

In this study, a total of 39 non-redundant sequences were obtained by integrating the results of the BLAST and online software Pfam verification of the transcriptome database. The resulted sequences were then searched again using Pfam batch search and 27 *Aux/IAA* genes with confidant domain were confirmed as representatives of *BsIAA* gene family after a manual curation. For the convenience of the study, the 27 *BsIAAs* were sequentially designated according to their sequence length, from *BsIAA1* to *BsIAA27* (Table 2). Information about these *BsIAAs* is listed in Table 1, including gene name, locus ID, ORF length, and predicted characteristics of corresponding proteins. The length of the predicted *BsIAAs* ranged from 1010 bp (*BsIAA27*) to 4335 bp (*BsIAA1*), with molecular weights ranging from 88.94245 kDa to 362.22076 kDa, and the deduced isoelectric points varied widely, from 4.73 (*BsIAA1*&*2*) to 5.17 (*BsIAA27*). The instability index analysis found that except the proteins of *BsIAA3, 4, 13, 16, 17, 26, 27* were stable (unstable index < 40), the rest were unstable proteins (unstable index > 40). Subcellular localization analysis was detected to localize to the Nucleus Secondary structure analysis found that proteins of *BsIAA* family accounted for a large proportion of random coils, of which 26 were the largest proportion of random coils.

**Table 2.** Detailed information about 27 predicted Aux/IAA proteins of *B. striata.*

Gene name	Genes ID in the databases	Aux/IAA ORF	Peptide lengths	isoelectric point	Instability index	subcellular localization	Alpha helix％	Extended strand％	Beta turn％	Random coil％
*BsIAA1*	DN6980_c0_g1_i4	3479	1159	4.73	47.29	Nucleus	32.79	15.96	5.61	45.64
*BsIAA2*	DN6980_c0_g1_i7	3284	1094	4.73	46.72	Nucleus	31.99	16.09	5.76	46.16
*BsIAA3*	DN15793_c0_g1_i3	2915	971	4.83	38.41	Nucleus	29.35	17.82	6.90	45.93
*BsIAA4*	DN36550_c0_g1_i1	2794	924	4.83	38.76	Nucleus	30.74	14.18	4.33	50.76
*BsIAA5*	DN10105_c0_g2_i1	2561	853	4.80	49.78	Nucleus	19.11	15.12	3.52	62.25
*BsIAA6*	DN4720_c0_g2_i4	2555	851	4.82	43.30	Nucleus	19.51	14.57	4.35	61.57
*BsIAA7*	DN2432_c0_g1_i1	2531	843	4.82	46.33	Nucleus	20.52	15.07	3.91	60.50
*BsIAA8*	DN4720_c0_g1_i7	2510	836	4.83	46.84	Nucleus	19.02	15.91	4.31	60.77
*BsIAA9*	DN2018_c0_g1_i1	2426	808	4.80	42.61	Nucleus	27.72	15.22	4.08	52.97
*BsIAA10*	DN1124_c0_g3_i7	2072	690	4.80	41.21	Nucleus	19.86	15.80	4.78	59.57
*BsIAA11*	DN27_c0_g2_i1	2054	684	4.83	50.68	Nucleus	23.10	16.52	4.68	55.70
*BsIAA12*	DN1124_c0_g2_i1	2039	679	4.76	49.39	Nucleus	18.11	14.87	4.71	62.30
*BsIAA13*	DN27_c0_g1_i3	1964	654	4.91	35.94	Nucleus	18.50	16.51	5.20	59.79
*BsIAA14*	DN23527_c0_g2_i2	1676	588	4.93	46.43	Nucleus	15.23	13.08	3.58	68.10
*BsIAA15*	DN14453_c0_g1_i1	1379	459	4.87	47.15	Nucleus	16.78	15.90	2.61	64.71
*BsIAA16*	DN3142_c0_g1_i5	1004	334	5.05	38.03	Nucleus	26.95	13.17	4.49	55.39
*BsIAA17*	DN3142_c0_g2_i1	995	331	5.02	37.95	Nucleus	20.24	13.90	3.32	62.54
*BsIAA18*	DN6037_c0_g2_i2	893	297	5.03	41.89	Nucleus	17.17	17.17	3.37	62.29
*BsIAA19*	DN24793_c1_g1_i1	887	295	5.03	47.74	Nucleus	23.05	15.25	4.07	57.63
*BsIAA20*	DN47023_c1_g1_i2	866	288	4.97	58.98	Nucleus	17.71	17.71	2.43	62.15
*BsIAA21*	DN10844_c0_g2_i1	863	287	5.04	40.94	Nucleus	21.95	14.98	3.14	59.93
*BsIAA22*	DN6037_c0_g2_i3	851	283	5.03	42.38	Nucleus	17.67	16.61	3.18	62.54
*BsIAA23*	DN11321_c0_g1_i1	635	211	4.94	55.13	Nucleus	20.85	19.43	3.79	55.92
*BsIAA24*	DN67702_c0_g1_i1	536	178	4.98	47.64	Nucleus	23.03	19.10	7.30	50.56
*BsIAA25*	DN3142_c0_g3_i1	335	111	5.11	46.89	Nucleus	50.45	15.32	9.91	24.32
*BsIAA26*	DN4243_c0_g1_i3	254	84	4.97	37.69	Nucleus	29.76	20.24	7.14	42.86
*BsIAA27*	DN3624_c0_g2_i1	209	69	5.17	22.88	Nucleus	30.43	21.74	10.14	37.68

### 2.3 *Comparative phylogenetic analysis of* BsIAA

**Figure 3.**
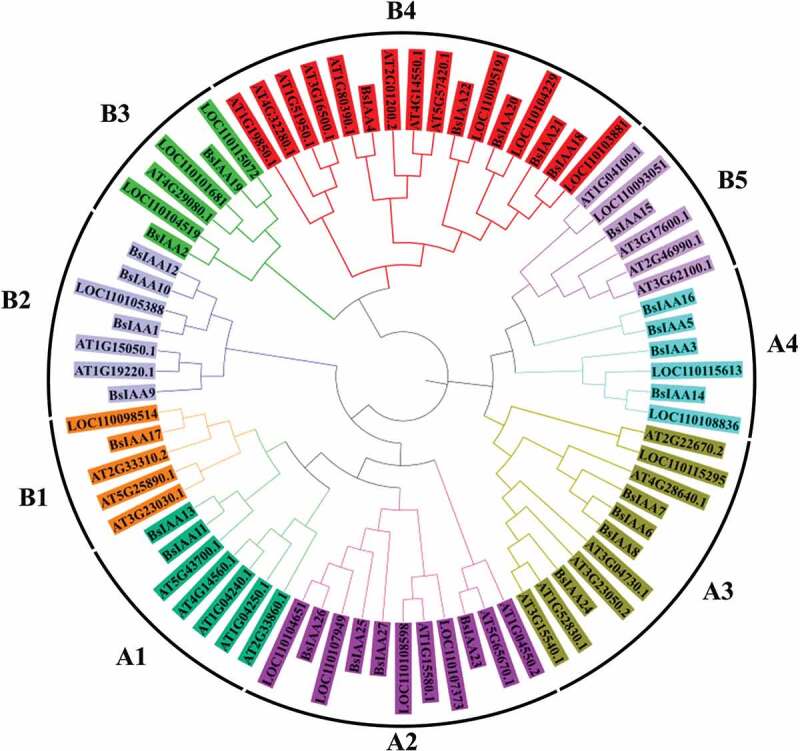
Phylogeny of Aux/IAA proteins from *B. striata, A. thaliana* and *D. officinale.*

To examine the evolutionary relationships among the *Aux/IAA* genes from *B. striata, D. officinale* and *A. thaliana*, a rooted phylogenetic tree was generated based on the alignment of amino acid sequences for 75 Aux/IAA proteins, including 27 *BsIAAs*, 16 *DoIAAs* and 32 *AtIAAs* (Figure 3). Phylogenetic distribution indicated that Aux/IAA proteins can be classified into two major groups (Group A and Group B), which could be further subdivided into four (A1-A4) and five (B1-B5) subgroups, respectively. Among them, group A and B consisted of 35 and 40 Aux/IAA proteins, respectively. A sister pair indicates the closest relatives within a phylogenetic tree. Within this tree, a total of 25 sister pairs were found, consisting of 10 and 15 pairs in group A and B. This pattern of two major groups for *Aux/IAA* gene family members in the phylogenetic tree was similar to that reported for other plants including rice [22], Moso bamboo (*Phyllostachys pubescens*) [23], soybean [12] and *Brassica napus* [2], which suggested that the *Aux/IAA* genes have been widely conserved in different groups.

**Figure 4.**
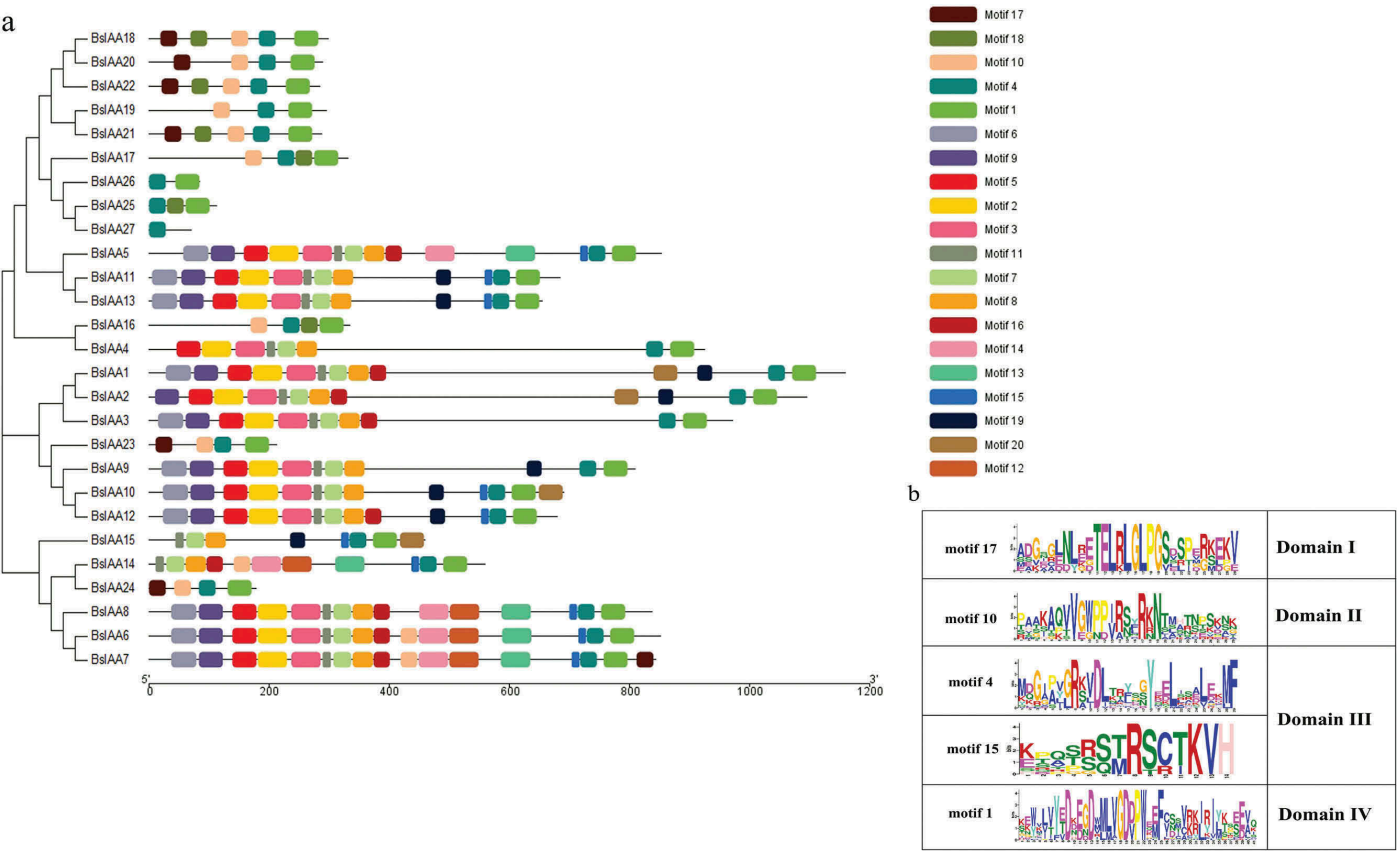
Phylogenetic relationships and protein motif distribution analysis.a. The phylogenetic tree of the *BsIAAs*. b. Motif distributions in the *B. striata.*

Meanwhile, to gain a better understanding of the structural diversity of the *BsIAAs*, we also built a separate phylogenetic tree using the same method (Figure 4). Four typical domains were detected among *BsIAAs*, similar to the proteins of *AtIAAs* (Figure 1). MEME analysis found that four conserved domains (Domain I to Domain IV) of *BsIAAs* protein were contained in five motifs (motif 1, 4, 10, 15 and 17). Except *BsIAA27*, the others all contained motif 1 and motif 4. Most *BsIAA*s did not contain motif 17, and *BsIAA6, 7, 16* to *24* contained motif 10 but no Domain II. Combined with the phylogenetic tree (Figure 3) showed that the similar branches and types of Aux/IAA protein motifs were identical or similar to each other.

### 2.4 *Polymorphism detection of EST-SSR in* BsIAAs

**Figure 5.**
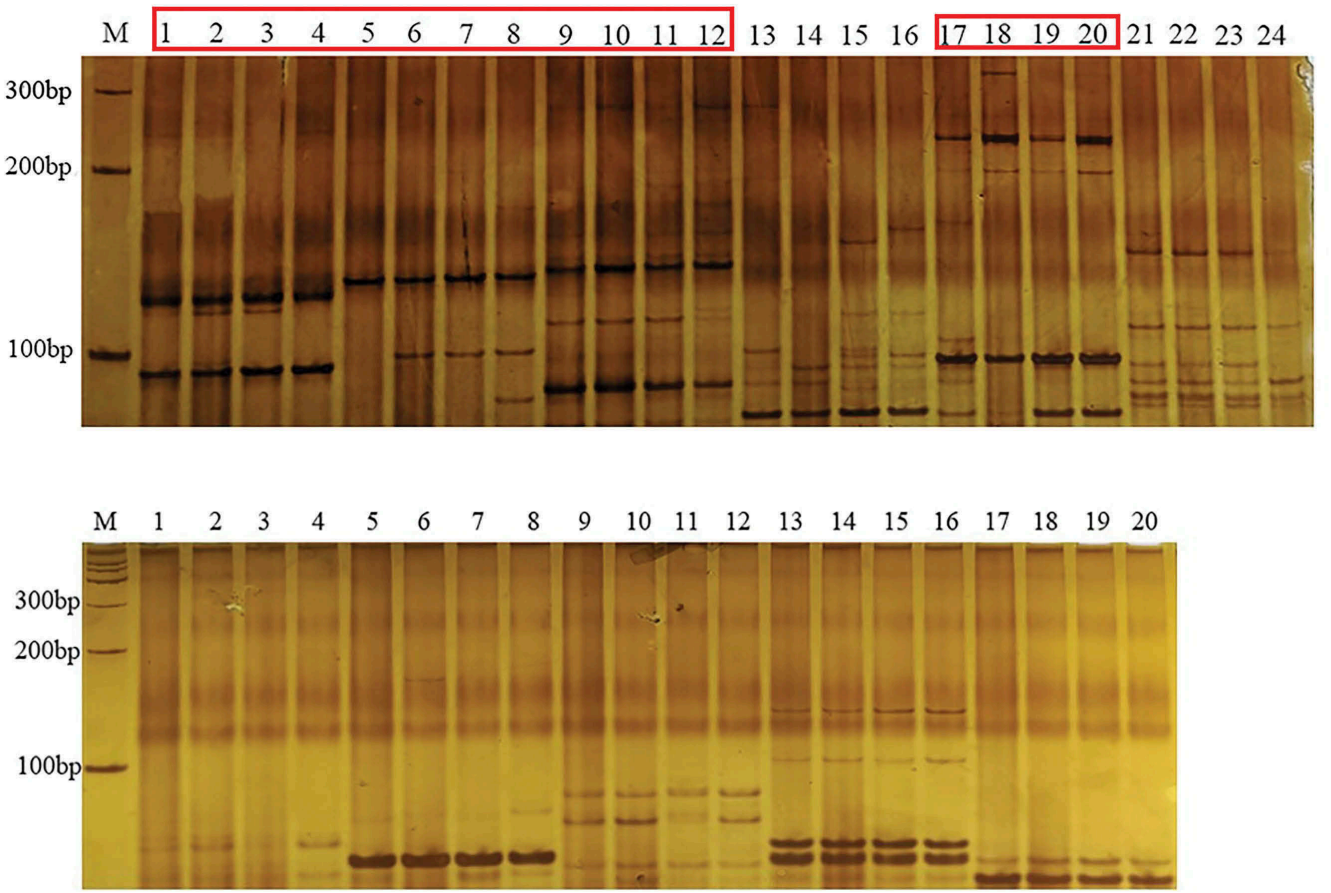
SSR profiles of *B. striata.*

In this study, 4 strains of *B. striata* genomic DNA were amplified with the designed 11 pairs of primers (Table 3). Among them, four pairs of primers can be amplified stably, and the length of the amplified product ranged from 70 to 300 bp (Figure 5). Four DNAs amplified different polymorphic bands, and the percentage of polymorphic loci was 30%, indicating that the *Aux/IAA* gene family in different regions was genetically conserved and also presented different polymorphisms. Thus, SSR primers can be used as molecular markers to identify different strains of the *Aux/IAA* gene family.

**Table 3.** The information of EST-SSR primers.

Gene name	Number of motifs	Forward primer	Tm (°C)	Reverse primer	Tm (°C)	Target band size (bp)
*BsIAA1*	(gca)8	TGATGCAGAACCAGCATGT	54.3	AGATGCAGCTGGTTGGTCT	56.7	150–238
*BsIAA2*	(gca)8	GAACCAGCATGTGATCAGG	53.8	TGAATCTGATGTTCGCTCAG	52	160–300
*BsIAA3*	(cag)5	CAGGGCGATTTAAACTGAA	50.1	TGCTGCTGCTGAATCTGATA	53.6	120–230
*BsIAA4*	(ggc)4	CGTGCTGTTTATTCGTGATG	51.6	GGTATACGCCGCTTTCTGAT	53.4	70–150
*BsIAA6*	(gcc)5	GGTGAACGTGGATCTGAAAG	53.4	ATTCATCCGCATGACGACG	55.6	112–170
*BsIAA8*	(gcc)5	GGTGAACGTGGATCTGAAAG	53.4	GCATTCATCCGCATGACGA	55.9	110–270
*BsIAA9*	(ggc)4	AGCGTGCTGTTTATTTGGAAC	53.5	CATATTTGCTCAGCGGAATC	51.2	160–210
*BsIAA17*	(ggc)7	GCTGGATCTGATTAGCATTG	50.7	CCAGTTCTTCTTCCACCAC	53.1	80–150
*BsIAA18*	(gcg)12	GAAGGCGAAATGGAAAAAGG	51.9	GTGCTCATGGAGTTTTTACG	51.3	210–300
*BsIAA21*	(gcg)5	AACGATAAATGGGGCTTTTG	50.5	GTGTTTTTACGATGGCTACG	51.3	70–220
*BsIAA22*	(ggc)7	GAAGGCGAAATGGAAAAAGG	51.9	CCATCCGCATCTTCTTTGTT	52.8	70–160

## 3 Discussion

Auxin is a key signaling molecule in the process of plant growth and development. As the earliest discovered plant hormone, its physiological role is extensive, affecting cell division, enlargement, and differentiation. Aux/IAA proteins have been suggested to bind with ARFs and prevent activation of auxin-responsive genes in the absence of auxin [8]. However, in *B. striata*, there was very little information about the *Aux/IAA* genes, until total of 27 *Aux/IAA* genes were identified in *B. striata* in this paper, though lower than 29 *Aux/IAA* genes in *A. thaliana* [24] and 31 *Aux/IAA* genes in *Oryza sativa* [22]. However, this study was based on the transcriptome data of the *B. striata*. Due to incomplete data, the identified *Aux/IAA* genes were very few, so the whole genome could be sequenced and a database could be established in the subsequent studies.

According to the physicochemical analysis of 27 proteins of *BsIAAs* family, most of them acted in an acidic subcellular environment, which means they were unstable proteins. About the secondary structures, the proportion of random coils was largest in most of Aux/IAA family proteins. Subcellular localization was localized in the nucleus, suggesting that the Aux/IAA protein might play a role in the nucleus.

By analyzing the conserved domains of *BsIAA* gene family proteins, we found that the *BsIAAs* contain four domains, namely Domain I, II, III, IV. In which the Domain I at the N terminus had three repeat leucine residues, referred to as ‘LxLxL’ motif (L refers to leucine, x means any amino acid residue), which was required for the transcriptional repression function of Aux/IAA protein [25], and this was the smallest and least strictly conserved among the conserved domains. Domain II contained a target site for ubiquitination degradation of Aux/IAA protein with the core sequence of VGWPP. The dominant mutation in this region made Aux/IAA protein unable to enter the ubiquitination pathway and lead to enhanced stability [26]. Domain III contained a β sheet and two α helices (α1 and α2), which played an important role in the dimerization of Aux/IAA protein [2]. Domain IV included the acidic region and the SV40 type NLS (PKKKRKV) [26]. These were similar to *A. thaliana* [27], *Oryza sativa* [22], *Zea mays. L* [11]., *Cucumis sativus* [10] and other *Aux/IAA* gene family, indicating that Aux/IAA protein had a high sequence conservation. There were multiple amino acid changes in the conserved domains of *BsIAA* proteins. This result was similar to the analysis of the *Brassica rapa* Aux/IAA proteins [14], indicating that the altered regions might have new functions or only some typical functions of Aux/IAA proteins.

According to the motif analysis, it indicated that these genes did not contain motif 17 might not be involved in classical auxin signal transduction. For genes containing motif 10, their protein life was longer than others [12]. The results indicated that the more frequent motif was an important conserved motif in the *Aux/IAA* domain. Online analysis of the less frequent motif by SMART had no relevant description of its functional annotations, so it required further investigation.

Based on the phylogenetic analysis in this study, *BsIAAs* were divided into two classes which contained 4 and 5 subfamilies, respectively. The similar branches of Aux/IAA proteins had the same or similar motifs, such as genes between *BsIAA10* and *BsIAA12, BsIAA11* and *BsIAA13, BsIAA18* and *BsIAA20*, indicating that Aux/IAA proteins were conserved, which was similar to the *Aux/IAA* family genes in *Medicago truncatula* [28] and *Brassica napus* [2]. The phylogenetic tree constructed from the Aux/IAA proteins of *B. striata, D. officinale* and *A. thaliana* showed that the *B. striata* Aux/IAA proteins had similar work to *D. officinale*, which indicated that *BsIAA* and *DoIAAs* were relatively close to each other in evolutionary relationship. This analysis could be used for further exploring the protein functions of *BsIAA*s.

Molecular markers are excellent tools to study the genetic relationships and genomic evolution of species. Due to the conserved nature of flanking sequences, SSR markers developed in one species can be employed to detect these microsatellite loci in other related species [29]. In this study, EST-SSR site analysis was performed on *BsIAA*s, revealing that the ratio of polymorphic alleles was 30%, which means *B. striat*a in different regions have high genetic conservatism. And it was mainly due to the conservation of *Aux/IAA* gene itself, but it also showed the genetic diversity. Therefore, SSR molecular markers could be used to evaluate the genetic diversity in different regions. We identified the genetic relationship by using the amplified primers in different regions and number of bands, and estimate the genetic relationship in order to provide more accurate information for breeding, breed identification and genetic structure research.

Auxin plays a very important role in plants, affecting the yield and quality of *B. striata*. Based on the first transcriptome databases of *B. striata*, we identified the 27 members of the *Aux/IAA* gene family and analyzed their basic physicochemical properties, subcellular localization, protein conserved domains, conserved motifs and phylogenetics in this paper. Transcriptome gene expression characteristics of the group were comprehensively analyzed and SSR molecular markers were also performed. In conclusion, these genes can be divided into group A and group B, which contain 4 and 5 subfamilies, respectively. Among them, *BsIAAs* were more closer to those of *D. officinale* compared to *A. thaliana*. In this study, a total of 11 pairs of primers were designed, among which 4 pairs could be amplified stably and showed different polymorphism. These results laid a solid foundation for further study of the biological functions of *BsIAA*s, as well as the identification of *B. striata* germplasm resources and the analysis of phylogenetic relationships.

## References

[1] Yang F, Song Y, Yang H, et al. An auxin-responsive endogenous peptide regulates root development in *Arabidopsis*. J Integr Plant Biol. 2014;56(7):635–647. 24479837 10.1111/jipb.12178

[2] Li H, Wang B, Zhang Q, et al. Genome-wide analysis of the auxin/indoleacetic acid (*Aux/IAA*) gene family in allotetraploid rapeseed (*Brassica napus L*.). BMC Plant Biol. 2017;17(1):204–213. 29145811 10.1186/s12870-017-1165-5 PMC5691854

[3] Kepinski S, Leyser O. Auxin-induced SCFTIR1-Aux/IAA interaction involves stable modification of the SCFTIR1 complex. Proc Nat Acad Sci. 2004;101(33):12381–12386. 15295098 10.1073/pnas.0402868101 PMC514484

[4] Hu W, Zuo J, Hou X, et al. The auxin response factor gene family in banana: genome-wide identification and expression analyses during development, ripening, and abiotic stress. Front Plant Sci. 2015;6(undefined):742–757. 26442055 10.3389/fpls.2015.00742 PMC4569978

[5] Hayashi K. The interaction and integration of auxin signaling components. Plant Cell Physiol. 2012;53(6):965–975. 22433459 10.1093/pcp/pcs035

[6] Li F, Wu M, Liu H, et al. Systematic identification and expression pattern analysis of the Aux/IAA and ARF gene families in moso bamboo (*Phyllostachys edulis*). Plant Physiol Biochem. 2018;130:431–444. 30077919 10.1016/j.plaphy.2018.07.033

[7] Rybel BD, Vassileva V, Parizot B, et al. A novel Aux/IAA28 signaling cascade activates GATA23-dependent specification of lateral root founder cell identity. Curr Biol. 2010;20(19):1697–1706. 20888232 10.1016/j.cub.2010.09.007

[8] Luo J, Zhou JJ, Zhang JZ. Aux/IAA gene family in plants: molecular structure, regulation, and function. Int J Mol Sci. 2018;19(1):259–274. 10.3390/ijms19010259 PMC5796205 29337875

[9] Yu H, Soler M, San CH, et al. Comprehensive genome-wide analysis of the *Aux/IAA* gene family in *Eucalyptus*: evidence for the role of EgrIAA4 in wood formation. Plant Cell Physiol. 2015;56(4):700–714. 25577568 10.1093/pcp/pcu215

[10] Gan D, Zhuang D, Ding F, et al. Identification and expression analysis of primary auxin-responsive Aux/IAA gene family in cucumber (*Cucumis sativus*). J Genet. 2013;92(3):513–521. 24371172 10.1007/s12041-013-0306-3

[11] Wang Y, Deng D, Bian Y, et al. Genome-wide analysis of primary auxin-responsive *Aux/IAA* gene family in maize (*Zea mays. L*.). Plos One. 2010;37(8):3991–4001. 10.1007/s11033-010-0058-6 20232157

[12] Singh VK, Jain M. Genome-wide survey and comprehensive expression profiling of *Aux/IAA* gene family in chickpea and soybean. Front Plant Sci. 2015;6:918–932. 26579165 10.3389/fpls.2015.00918 PMC4621760

[13] Ke Y, Abbas F, Zhou Y, et al. Genome-wide analysis and characterization of the *Aux/IAA* Family genes related to floral scent formation in *hedychium coronarium*. Int J Mol Sci. 2019;20(13):3235–3255. 10.3390/ijms20133235 PMC6651449 31266179

[14] Paul P, Dhandapani V, Rameneni JJ, et al. Genome-wide analysis and characterization of *Aux/IAA* family Genes in *Brassica rapa*. PLoS One. 2016;11(4):e0151522. 27049520 10.1371/journal.pone.0151522 PMC4822780

[15] Lingna C, Xianggan Z, Xiaojuan G, et al. The roles of *Aux/IAA* gene family in development of *Dendrocalamus sinicus* (Poaceae: bambusoideae) inferred by comprehensive analysis and expression profiling. Mol Biol Rep. 2019;46:1625–1634. 30690658 10.1007/s11033-019-04611-2

[16] Chen Z, Cheng L, He Y, et al. Extraction, characterization, utilization as wound dressing and drug delivery of *Bletilla striata* polysaccharide: A review. Int J Biol Macromol. 2018;120:2076–2085. 30195614 10.1016/j.ijbiomac.2018.09.028

[17] Xu DL, Chen HB, Aci M, et al. De Novo assembly, characterization and development of EST-SSRs from *Bletilla striata* transcriptomes profiled throughout the whole growing period. PLoS One. 2018;13(10):e0205954. 30365506 10.1371/journal.pone.0205954 PMC6203367

[18] Kumar R, Tyagi AK, Sharma AK, et al. Genome-wide analysis of auxin response factor (*ARF*) gene family from tomato and analysis of their role in flower and fruit development. Mol Genet Genomic. 2011;285(3):245–260. 10.1007/s00438-011-0602-7 21290147

[19] Huang X, Bao YN, Wang B, et al. Identification and expression of *Aux/IAA, ARF*, and *LBD* family transcription factors in *Boehmeria nivea*. BMC Plant Biol. 2016;60(2):244–250.

[20] Liu H, Wu M, Zhu D, et al. Genome-Wide analysis of the *AAAP* gene family in moso bamboo (*Phyllostachys edulis*). BMC Plant Biol. 2017;17(1):29–46. 28143411 10.1186/s12870-017-0980-z PMC5282885

[21] Aboul-Maaty NAF, Oraby HAS. Extraction of high-quality genomic DNA from different plant orders applying a modified CTAB-based method. Bull National Res Centre. 2019;43(1). DOI:10.1186/s42269-019-0066-1

[22] Jain M, Kaur N, Garg R, et al. Structure and expression analysis of early auxin-responsive *Aux/IAA* gene family in rice (*Oryza sativa*). Funct Integr Genomics. 2006;6(1):47–59. 16200395 10.1007/s10142-005-0005-0

[23] Wang W, Gu L, Ye S, et al. Genome-wide analysis and transcriptomic profiling of the auxin biosynthesis, transport and signaling family genes in moso bamboo (*Phyllostachys heterocycla*). BMC Genomics. 2017;18(1):870–885. 29132316 10.1186/s12864-017-4250-0 PMC5683460

[24] Fattorini L, Ronzan M, Piacentini D, et al. Cadmium and arsenic affect quiescent centre formation and maintenance in *Arabidopsis thaliana* post-embryonic roots disrupting auxin biosynthesis and transport. Environ Exp Bot. 2017;144:37–48.

[25] Li H, Tiwari SB, Hagen G, et al. Identical amino acid substitutions in the repression domain of auxin/indole-3-acetic acid proteins have contrasting effects on auxin signaling. Plant Physiol. 2011;155(3):1252–1263. 21252300 10.1104/pp.110.171322 PMC3046583

[26] Guilfoyle TJ, Hagen GJ. Getting a grasp on domain III/IV responsible for auxin response factor–IAA protein interactions. Plant Sci. 2012;190(3):82–88. 22608522 10.1016/j.plantsci.2012.04.003

[27] Carrier DJ, Bakar NT, Swarup R, et al. The binding of auxin to the *Arabidopsis* auxin influx transporter AUX1. Plant Physiol. 2008;148(1):529–535. 18614710 10.1104/pp.108.122044 PMC2528085

[28] Shen C, Yue R, Yang Y, et al. Genome-wide identification and expression profiling analysis of the *Aux/IAA* gene family in *Medicago truncatula* during the early phase of Sinorhizobium meliloti infection. PLoS One. 2014;9(9):e107495. 25226164 10.1371/journal.pone.0107495 PMC4166667

[29] Thakur AK, Singh KH, Singh L, et al. SSR marker variations in Brassica species provide insight into the origin and evolution of *Brassica* amphidiploids. Hereditas. 2018;155(6). DOI:10.1186/s41065-017-0041-5 PMC5516320 28729817

